# Urea cycle defects in adulthood: clinical presentation, diagnosis and treatment in genetically encoded hepatic metabolic disorders with a potential for encephalopathy

**DOI:** 10.1007/s11011-025-01619-5

**Published:** 2025-04-26

**Authors:** Anibh Martin Das

**Affiliations:** 1 Department of Paediatric Kidney, Liver and Metabolic Diseases, Hannover Medical School, Hannover, Germany; 2 Hannover Medical School, Carl Neuberg Str. 1, D- 30625 Hannover, Germany

**Keywords:** Urea cycle defect, Ammonia, Amino acids, Benzoate, Scavengers, Glycerophenylbutyrate

## Abstract

Hyperammonaemia is an important cause for encephalopathy. Ammonia is the waste product of amino acid degradation and cannot be excreted via urine. Ammonia is metabolized to water-soluble urea via the urea cycle. Hyperammonaemia not only occurs during acute liver failure, but also in rare genetically determined defects of enzymes or transporters involved in the urea cycle resulting in elevated ammonia concentrations. Enzyme defects include deficiency of carbamylphosphate synthase, N-acetylglutamate synthase, ornithine transcarbamylase, argininosuccinate lyase and arginase, transporter defects are citrin deficiency and HHH-syndrome. These urea cycle defects (UCD) mostly manifest for the first time during the neonatal period, infancy or childhood, however first clinical manifestations including encephalopathy may be observed in adulthood in milder forms. Therefore, physicians treating adults should be aware of clinical symptoms in UCD to make a timely diagnosis and initiate treatment. In adulthood, clinical symptoms are often uncharacteristic including headache, avoidance of high-protein food, psychiatric symptoms triggered by heavy exercise or delivery of a child, autism, attention deficit, lethargy, developmental delay and epilepsy. Elevated ammonia concentrations in blood are the biochemical hallmark. Some UCDs can be diagnosed at metabolite level, others only at genetic level. Treatment consists of eucaloric, low-protein diet supplemented with essential amino acids and vitamins/trace elements, and intake of arginine or citrulline. Pharmacological scavengers of nitrogen are benzoate and butyrate. If conservative therapy fails, hemodialysis should be considered. Prompt treatment during acute crises is essential for optimal outcome. Liver transplantation is considered in metabolically unstable patients. For arginase deficiency, enzyme replacement therapy is available.

## Introduction

**Fig. 1:**
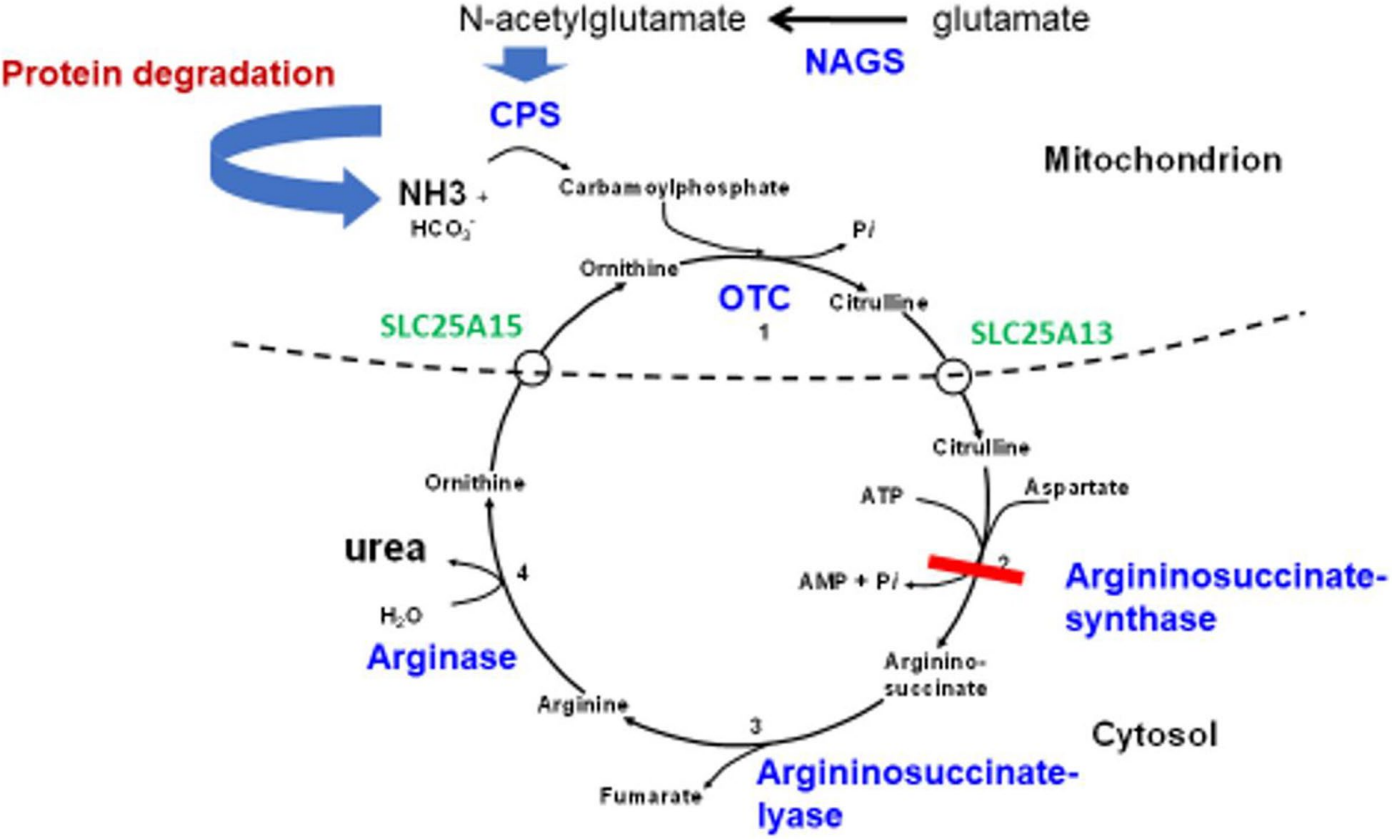
Schematic view of the urea cycle. Enzymes, transporters and metabolites involved in the urea cycle. Ammonia resulting from protein degradation is converted to urea. As an example of a UCD, enzyme deficiency of ASS in ASSD is marked by the red bar

Urea cycle defects (UCDs) are a group of rare inborn errors of metabolism with a cumulative incidence of 1:35,000–1:69,000 (Häberle et al. 2019; Summar et al. 2013). This group includes the 4 ‘classical’ UCDs with deficiency/dysfunction of the following enzymes: carbamyl-(carbamoyl-) phosphate synthase 1 (CPS 1), ornithine transcarbamylase (OTC), argininosuccinate synthase (ASS), and the less common N-acetylglutamate synthase (NAGS) (see Fig. 1). Enzyme deficiencies of argininosuccinate lyase (ASL) and arginase (ARG1) have a more chronic course with different symptoms. Furthermore, transporter deficiencies can cause UCDs, namely the ornithine/citrulline antiporter (ORNT1, SLC25A15) and the aspartate/glutamate antiporter (citrin, SLC25A13) catalysing amino acid transport from the mitochondrial matrix to the cytosol and vice versa (Fig. 1). UCDs are characterised by compromised metabolism of neurotoxic ammonia to water-soluble urea, which can be excreted via urine. Under physiological conditions, ammonia in blood is predominantly converted to a cation (NH4^+^), which cannot be excreted by the kidneys in large quantity. The biochemical hallmark is hyperammonaemia, specific metabolites can be found in some subtypes of UCD. Most UCDs are inherited as autosomal-recessive traits with the exception of OTC-deficiency (OTCD) which is inherited in an X-linked manner.

In many countries, UCDs are not target diseases of the newborn mass screening programme, thus diagnosis has to be based on clinical suspicion and/or suspicious laboratory parameters like elevation of ammonia and glutamine (as a long-term parameter for hyperammonaemia) or reduced urea concentrations in blood. Alternatively, an index case in the family may prompt the diagnostic workup. Adult patients with UCD may have been already diagnosed and treated during childhood and have to transition from paediatrics to adult medicine, some milder forms of UCDs may have their first clinical manifestation during adulthood triggered by catabolism or an excessive protein load. For the latter group, awareness of adult physicians, like neurologists, psychiatrists and general practitioners, regarding clinical symptoms of UCDs is essential to make a timely diagnosis. Hyperammonaemia is an emergency situation which requires giving enough calories to avoid catabolism, start nitrogen scavengers and supplement arginine.

The aim of the present manuscript is to make clinicians aware of possible symptoms, laboratory findings and treatment options in adult patients with UCDs. The focus is not on pathophysiological processes resulting in neurological symptoms which can be found elsewhere (e.g. Braissant 2010; Braissant et al. 2013; Waisbren et al. 2016).

## General (generic) clinical symptoms in UCDs

Symptoms may be variable depending on the severity of the biochemical defect and the specific subtype of UCD (e.g. Häberle et al. 2019). UCDs are often regarded as diseases manifesting during childhood, however first manifestations even occur during adolescence and adulthood in some patients (Nassogne et al. 2005; Summar et al. 2008). For timely diagnosis and treatment in adults, awareness of clinical symptoms of UCDs amongst clinicians, especially neurologists, but general practitioners and psychiatrists as well, is essential.

In the most severe neonatal form, anorexia, vomiting, lethargy, coma, epileptic seizures, central hypothermia as well as tachydyspnea are observed. These symptoms are triggered by postpartal/neonatal catabolism leading to the mobilisation of amino acids from endogenous sources.

On the other end of the spectrum, patients with the infantile or adult forms of UCDs may manifest with developmental delay, avoidance of high-protein food like meat, frequent headache, attention deficit/poor concentration, psychiatric symptoms, autism or epilepsy. Less common symptoms in milder cases are episodic cortical visual loss (Prasun et al. 2015) and chorea (Häberle et al. 2019). Cerebral edema may be a complication of hyperammonaemic crises, which may be fatal (Maghmoul et al. 2024).

Metabolic decompensation in adults with coma and frequent epileptic seizures may be triggered by catabolic spells, for example during military service in males or pregnancy and delivery in females or intercurrent febrile illness (Summar et al. 2005; Smith et al. 2005). Chemotherapy (especially arginase therapy), steroid treatment and valproate treatment are other triggers of metabolic decompensation.

Specific symptoms unique to respective subtypes of UCDs can be found below (5. Specific UCDs).

## General (generic) laboratory findings in UCDs

Typical laboratory findings in UCDs include hyperammonaemia, low urea concentrations in blood and abnormal amino acid profiles in plasma which are specific for the single subtypes of UCDs (see below). Glutamine as a long-term parameter reflecting ammonia levels is often elevated. Organic acids in urine and an acylcarnitine profile in blood shall be ordered to exclude secondary metabolic causes of hyperammonaemia.

‘Secondary’ hyperammonaemia may be a feature in other metabolic disorders like organic acidurias (e.g. methylmalonic aciduria, propionic aciduria) (Ribas et al. 2022) or carbonic anhydrase 5A-deficiency (CA5A gene) (van Karnebeek et al. 2014; Diez-Fernandez et al. 2016). Valproic acid treatment may also lead to hyperammonaemia (Aires et al. 2011).

Hyperammonaemia is also found in liver failure as an unspecific finding in non-genetic diseases, which can play a role in hepatic encephalopathy.

Diagnostic aspects unique to specific subtypes are mentioned below (5. Specific UCDs).

## General (generic) treatment principles in UCDs

### Diet

Long-term treatment includes a low-protein diet to limit ammonia production which should contain enough calories to maintain anabolism. Special amino acid mixtures composed of essential amino acids supplemented with trace elements (e.g. iron, selenium, zinc, copper, magnesium) and vitamins (e.g. cobalamin) have to be given. The amount of natural protein has to be titrated individually based on ammonia and glutamine concentrations in plasma.

Acute hyperammonaemic decompensation may occur as the initial, neonatal manifestation of UCD or during catabolism induced by acute febrile illness in later life and requires swift treatment. The long-term cognitive outcome is correlated with the duration of the hyperammonaemic coma and the peak ammonia level (Posset et al. 2019; Häberle et al. 2019). To create anabolism a glucose infusion (10% w/v) supplemented with electrolytes has to be started immediately. If hyperglycaemia develops, insulin may be added. Small amounts of protein (0.25 g/kg per day) should be started after 2 days of glucose infusion. If hyperammonaemia cannot be controlled by glucose infusion and medication (see 4.2) hemodialysis should be considered (see 4.3). Therefore, patients in a hyperammonaemic crisis should be urgently transferred to a specialist centre where hemodialysis is available (e.g. Häberle et al. 2019).

### Pharmacological treatment

At physiological pH, blood ammonia is converted to a cation which the kidneys can only poorly excrete. The main function of the urea cycle is to metabolize ammonia to urea which is water-soluble and can readily be excreted via urine. The scavengers allow elimination of nitrogen independently of the dysfunctional urea cycle.

**Fig. 2:**
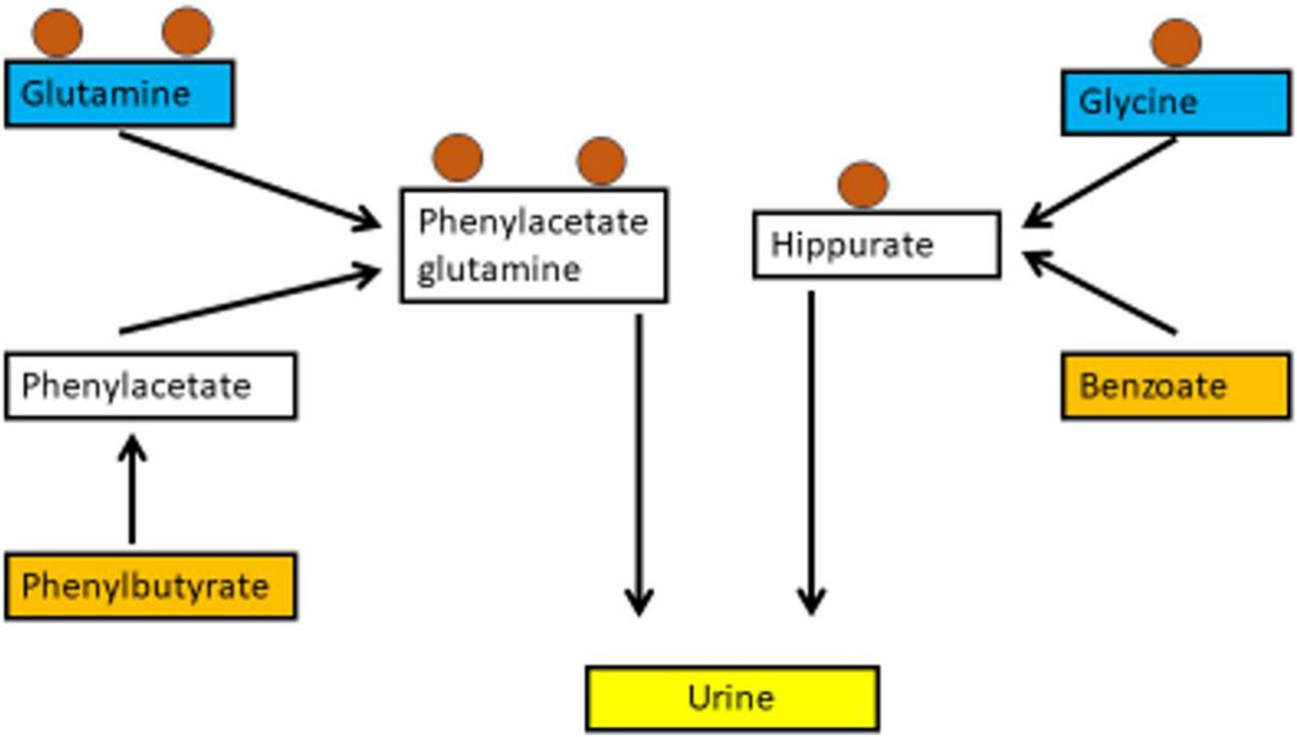
Schematic view of nitrogen scavengers. The mode of action of the nitrogen scavengers phenylbutyrate (left) and benzoate (right). Both compounds are conjugated with amino acids containing nitrogen atoms (circles) and form water-soluble compounds, which can be excreted via urine

To eliminate nitrogen in patients with UCDs, scavengers like benzoate or butyrate are used. (e.g. Ah Mew et al. 2020; Häberle et al. 2019). Benzoate is conjugated with the amino acid glycine to hippurate, while the prodrug phenylbutyrate is first metabolised to phenylacetate in the liver and then conjugated with glutamine to yield phenylacetyglutamine, which is excreted by the kidneys (Fig. 2). There are 2 preparations of phenylbutyrate on the market. Glycerophenylbutyrate is an oily suspension without taste, while sodium phenylbutyrate is a powder with an unpleasant taste frequently causing gastrointestinal symptoms which hampers adherence to medication. The uptake of glycerophenylbutyrate is slower, as the molecule has to be first digested by pancreas lipases to yield phenylbutyrate and glycerol, which results in favourable pharmacokinetics (Monteleone et al. 2013).

The dose for both substances is 250–500 mg/kg per day, the maximum dose is 12 g per day. The scavengers should be taken with meals. The oral maintenance dose is titrated individually based on plasma ammonia levels and glutamine concentration in plasma.

Arginine becomes a semi-essential amino acid in most UCDs and therefore has to be supplemented, again the maximal dose is 12 g per day. The maintenance dose is titrated individually based on plasma arginine levels which should be in the reference range.

During intercurrent febrile illness, an emergency regimen with reduced amounts of natural protein supplemented with high amounts of protein-free high-calorie food shall be promptly started at home. During acute metabolic decompensation, scavengers and arginine may be given intravenously, the dosage is the same as orally but sometimes has to be increased based on catabolism with elevated ammonia concentrations.

Benzoate treatment may be given to the pregnant females if prenatal diagnosis of an UCD has been established, in an attempt to load the foetus prenatally (Das et al. 2009).

If a female patient with UCD is giving birth to a child, precautions have to be taken to avoid catabolism and benzoate and arginine infusion may be given.

### Hemodialysis

If dietary and pharmacological treatment are not able to control acute hyperammoanemia, extracorporeal detoxification shall be rapidly initiated (Häberle et al. 2019).

Hemodialysis is the most efficient modality of extracorporeal detoxification. Continuous veno-venous hemofiltration or even peritoneal dialysis are less efficient ways of ammonia extraction.

Dialysis may induce catabolism, therefore enough glucose, fatty acids and small amounts of protein (e.g. 0.25 g/kg per day) shall be provided to support anabolism. Involvement of a specialised metabolic centre at an early stage of decompensation and timely transfer of the patient to a centre where extracorporeal detoxification is available is recommended.

### Liver transplantation

If UCD-patients remain instable in terms of metabolic decompensations, orthotopic liver transplantation should be considered (Ah Mew et al. 2017; Häberle et al. 2019). Mostly, this is performed during infancy or childhood, however some patients suffering from UCD may become unstable during adolescence/early adulthood when growth stops. With respect to long waiting lists in adulthood, livers from living donors (carriers of genetic variants encoding for UCD enzymes) have been successfully transplanted (Kasahara et al. 2023).

If liver transplantation is successful, no diet and scavengers are needed. Liver transplantation is mostly successful in UCD-patients (Kido et al. 2021). As part of the urea cycle is also located in extrahepatic tissues like the intestine, often arginine supplementation is still required.

Gene therapy is under development (see 5.) and may replace liver transplantation. In view of this future option, some clinicians are reluctant to offer liver transplantation unless there is frequent metabolic decompensation.

Some subtypes of UCDs are amenable to specific therapies which are mentioned below (5. Specific UCDs).

## Specific UCDs

### Carbamyl (or Carbamoyl) phosphate synthase 1 (CPS1)-deficiency (*CPS1D*) and N-acetylglutamate synthase (NAGS)-deficiency (*NAGSD*)

Both disorders are inherited as autosomal-recessive traits.

NAGS catalyses the synthesis of N-acetylglutamate (NAG) which is an activator of CPS1 (Martínez et al. 2010). Most patients show symptoms during the neonatal period or in early infancy (Ah Mew et al. 2017; Häberle et al. 2019), however milder forms may manifest during adolescence (Wang et al. 2023).

Abnormal laboratory values include elevated ammonia levels and glutamine concentration in blood while arginine and citrulline are typically low. Orotic acid in urine and blood is negative. As the amino acid profile in plasma does not show a specific pattern, genetic analysis is required for establishing the diagnosis of CPS1- and NAGS-deficiency. Nowadays, enzyme testing in liver tissue is rarely indicated, however this may be helpful when genetic testing is inconclusive.

Treatment consists of the general measures described above.

In NAGS-deficiency, oral carbamylglutamate (carglumic acid) can be given to activate CPS1, which results in increased protein tolerance (Singh et al. 2024). In CPS-deficiency, liver transplantation is often considered due to metabolic instability.

### Ornithine transcarbamylase (OTC)-deficiency (*OTCD*)

This disorder is inherited as an X-linked trait. Therefore, the clinical course is mostly severe in males, however attenuated forms are observed even in males. Most affected males show metabolic decompensation already in the neonatal period due to postnatal catabolism (Ah Mew et al. 2017; Häberle et al. 2019). In females, the clinical course is variable ranging from neonatal metabolic decompensation to first clinical symptoms during adulthood (Pizzi et al. 2019; Nambiar et al. 2022). The clinical course in females depends on Lyonisation of the X-chromosomes, resulting in a mild course when the affected X-chromosome is inactivated or severity comparable to male patients when the unaffected X-chromosome is inactivated.

The amino acid profile in plasma is mostly unrevealing, only showing unspecific glutamine elevation. Orotic acid in urine and blood may be elevated. For a definite diagnosis molecular genetic analysis is required.

Treatment includes protein-reduced diet, use of scavengers and citrulline (preferred to arginine) supplementation. In male OTC-patients, liver transplantation is often considered due to metabolic instability of patients.

### Argininosuccinate synthase (ASS)-deficiency (citrullinaemia 1; *ASSD*)

Citrullinaemia follows autosomal-recessive inheritance. Clinical manifestation is as described above. Most patients show their first metabolic decompensation in the postnatal period, often with residual symptoms after re-compensation of catabolism (Ah Mew et al. 2017; Häberle et al. 2019).

Apart from the typical laboratory alterations described above, the specific biochemical hallmark of this disorder is an elevation of citrulline in blood. The disorder should be confirmed genetically.

Patients suffering from citrullinaemia are treated by a protein reduced diet, scavengers and arginine supplementation as detailed above.

### Argininosuccinate lyase (ASL)-deficiency (argininosuccinic aciduria; *ASLD*)

Patients suffering from this autosomal-recessive disorder are often clinically asymptomatic at birth, however some patients manifest with hyperammonaemic coma in the first days of life. The neurocognitive development is hampered in many patients later on in life despite prevention of hyperammonaemic crises (Baruteau et al. 2017). This may be due to chronic toxicity of argininosuccinate or accumulation of guanidino compounds (Diez-Fernandez et al. 2019). Thus, from a pathophysiological point of view chronic toxicity by argininosuccinate and other compounds overlaps with acute toxicity by hyperammonaemia. This is different from the pathophysiology of the other UCDs.

Biochemically, argininosuccinate is elevated in body fluids. Hyperammonaemia and glutamine elevation in plasma are not present in all patients. Molecular genetics shall be performed as confirmatory diagnostic test.

As argininosuccinate can be readily excreted in urine, the risk to develop hyperammonaemia is lower than in the other UCDs. Many patients only need arginine supplementation, no scavengers are required, however others require diet, scavengers and arginine supplementation. Liver transplantation has been performed in some patients in an attempt to lower argininosuccinate levels. Neurocognitive outcome is variable, there is still extrahepatic production of argininosuccinate after transplantation (Yu et al. 1995).

### Arginase 1-deficiency (argininaemia; *ARG1D*)

The incidence of this condition is about 1:800,000. The clinical course of arginase 1-deficiency differs considerably from the other ‘classical’ UCDs (Schlune et al. 2015; Bin Sawad et al. 2022; Nteli et al. 2024). Most children do not have symptoms at birth or in the first few months of life in this autosomal-recessive disorder. Also, clinical symptoms are quite different from the other UCDs. Patients with arginase 1-deficiency develop progressive spastic paraplegia, affecting primarily the lower extremities, with developmental delay and seizures. Abnormal gait may result from spasticity. The risk of hyperammonaemia is lower than in the ‘proximal’ UCDs as argininosuccinate accumulates which is readily excreted via urine.

The biochemical hallmark is the accumulation of arginine in blood and other body fluids. Secondarily, production of guanidinoacetate, a neurotoxic compound, which also accumulates in guanidinoacetate-methyltransferase (GAMT) deficiency, is observed which may trigger epileptic seizures (Amayreh et al. 2014). Ammonia and glutamine concentrations in plasma are often normal.

Therapy classically consists of a low-protein diet supplemented with essential amino acids. This is rarely sufficient to normalize arginine levels. Attempts to reduce guanidinoacetate levels have been successful in a few ARG1D patients (Amayreh et al. 2014). Recently, pegzilarginase (Loargys^®^) has been approved in Europe as an enzyme replacement therapy which is able to normalize arginine concentrations in blood (Diaz et al. 2021; Russo et al. 2024). If diagnosis is made early in life, this therapy may be able to prevent neurological symptoms. Long-term observations are necessary to evaluate the impact of pegzilarginase on the clinical outcome. Neonatal screening would be necessary for early diagnosis and treatment in most patients where there is no index case in the family.

### HHH syndrome (hyperammonaemia, hyperornithineaemia, homocitrullinuria; *SLC25A15*)

Biochemically, this mitochondrial transporter defect (Fig. 1) is characterised by hyperammonaemia, hyperornithinaemia and homocitrullinuria, orotic acid may be increased in blood and urine. The underlying biochemical defect is ORNT1 deficiency. The clinical course differs from other UCDs. Apart from the typical symptoms observed in ‘proximal’ UCDs, patients develop spastic paraparesis, which occurs later in life comparable to ARG1D. This is preceded by pyramidal symptoms like hyperreflexia (Wild et al. 2019; Camacho et al. 2012). Hepatopathy is another clinical hallmark of this disorder and can manifest as acute liver failure (Fecarotta et al. 2006; Filosto et al. 2013). Diagnosis may be confirmed by mutation analysis.

Treatment consists of a low-protein diet and citrulline/arginine supplementation, phenylbutyrate should be administered to avoid hyperammonaemia (Camacho et al. 2012).

### Citrin deficiency (citrullinaemia type 2; SLC25A13)

This disease has a high prevalence (1:17,000) in Japan, but is also found in other areas of the world. It can manifest in neonates with (transient) intrahepatic cholestasis, hepatomegaly and failure to thrive, also symptomatic hypoglycaemia may be observed. In adults, recurrent encephalopathy, fatty liver, hepatoma, pancreatitis, craving for protein-rich food and aversion against carbohydrate-rich food may occur (Komatsu et al. 2023). Biochemical hallmarks are transient elevation of citrulline, threonine, methionine, tyrosine, hyperammonaemia and dylipidaemia in neonates, while citrulline elevation and hyperammonaemia are characteristic findings in adults (Saheki et al. 2004; Saheki and Song 2017).

Treatment consists of a galactose-free, MCT-enriched formula feeding, lipid-soluble vitamins should be substituted, carbohydrates should be avoided (Saheki and Song 2017).

## Conclusion

Hyperammonaemia is a common, unspecific feature of liver failure/liver dysfunction. Furthermore, hyperammonaemia is a biochemical hallmark of inborn errors of the urea cycle, the UCDs, and may lead to encephalopathy manifesting as coma and/or epilepsy. UCDs may be due to one of 6 enzymatic defects or one of 2 carrier/transporter defects. Catabolism is a common trigger of metabolic decompensation in UCDs. Severe UCDs mostly manifest during the neonatal period, infancy or childhood, milder forms may manifest with hyperammonaemic encephalopathy only in adolescence or adulthood, mostly triggered by catabolism.

Typical clinical symptoms are encephalopathy mediated by hyperammonaemia and developmental delay, feeding difficulties, headaches and psychiatric symptoms, which may be the first presentation of UCD. In arginase deficiency, spasticity of the lower limbs and gait abnormalities are typical clinical symptoms.

Diagnosis is based on the amino acid profile in plasma, orotic acid in blood or urine and organic acids in urine as well as mutation analysis.

Treatment consists of avoidance of catabolism and a low-protein diet supplemented with essential amino acids. Scavengers like benzoate and glycerophenylbutyrate can excrete nitrogen atoms independent of the dysfunctional urea cycle. Arginine may become a semi-essential amino acid, which has to be supplemented.

If dietary and pharmacological therapy fails, extracorporeal detoxification should be considered without delay and patients should be shifted to institutions where hemodialysis is available.

Arginase deficiency is the only UCD which can be treated by enzyme replacement therapy.

In those patients not sufficiently responding to pharmacological and dietary therapy, liver transplantation may be considered.

Patients with diagnosed UCDs should receive an emergency pass. Catabolism should be strictly avoided. Care has to be taken if elective surgery is planned, catabolism has to be avoided. Females with UCDs may develop hyperammonaemia after delivery, preventive measures have to be taken and ammonia should be closely monitored in the perinatal period.

## Future therapeutic options

Gene editing is a promising option to cure UCD-patients (Zabulica et al. 2021), gene therapy in UCD is another promising option (Duff et al. 2024).

Pharmacological hibernation has recently been described (Preußner et al. 2023) which may have a neuroprotective role in metabolic decompensation.

## Data Availability

No datasets were generated or analysed during the current study.

## Abbreviations

ARG1: Arginase 1
ASL: Argininosuccinate lyase
ASS: Argininosuccinate synthase
CA5A: Carbonic anhydrase 5A
CPS1: Carbamyl (Carbamoyl) phosphate synthase 1
D: Deficiency
GAMT: Guanidinoacetate-methyltransferase
HHH: Hyperammonaemia, Hyperornithinaemia, Homocitrullinuria
NAGS: N-acetylglutamate synthase
MCT: Medium chain triglycerides
ORNT1: Ornithine/citrulline antiporter
UCD: Urea cycle defect
